# Microsatellite-Primed PCR for Intra-species Genetic Relatedness in *Trichophyton ajelloi* Strains Isolated in Poland from Various Soil Samples

**DOI:** 10.1264/jsme2.ME13160

**Published:** 2014-05-24

**Authors:** Anita Ciesielska, Justyna Bohacz, Teresa Korniłłowicz-Kowalska, Paweł Stączek

**Affiliations:** 1 University of Łódź, Department of Microbial Genetics, ul. Banacha 12/16, 90–237 Łódź, Poland; 2 University of Life Sciences, Department of Environmental Microbiology, Laboratory of Mycology, ul. Leszczyńskiego 7, 20–069 Lublin, Poland

**Keywords:** *T. ajelloi*, soil, molecular differentiation, PCR melting profiles, microsatellite-primed PCR

## Abstract

*Trichophyton ajelloi* is a geophilic dermatophyte that specializes in the decomposition of native keratin. It exists in soil with a permanent influx of keratin matter. In the present study, two PCR-based methods were used for the identification and intra-species differentiation of *T. ajelloi* strains isolated from 3 types of soils with different physicochemical properties. The first method, employed for molecular identification, was PCR amplification of the 5.8S rRNA gene and its flanking regions encoding internal transcribed spacers (ITSs), followed by restriction enzyme digestion using endonuclease *Hin*fI. The second method, employed for molecular differentiation, was microsatellite-primed PCR (MSP-PCR) using the repetitive oligonucleotide (GACA)_4_. All the *T. ajelloi* strains were also identified using a traditional culture method. Our results showed that molecular identification using the PCR-restriction fragment length polymorphism (PCR-RFLP) method agreed with the identification made using the traditional approach. On the other hand, PCR-RFLP results showed no strain differentiation, while MSP-PCR using the (GACA)_4_ primer identified different varieties among the *T. ajelloi* strains. The reasons for the intra-species differentiation of *T. ajelloi* have been discussed.

Keratinophilic fungi are typically saprotrophs that live in natural environments on keratin structures such as hair, fingernails, feathers, and the epidermis. In terms of physiology, these are keratinolytic fungi, while “keratinophilicity” is an ecological equivalent of keratinolyticity (3, 16, 20, 21). The keratinolytic activities of keratinophilic fungi are known to vary (19). Kunert (21) proposed that keratinophilic fungi using more than 40% of native keratin during growth as the sole source of C, N, and energy after 8 weeks should be classified as strongly keratinolytic, while those that do not use more than 40% (even over a period longer than 8 weeks) should be considered as weakly keratinolytic. However, fungi that degrade less than 20% of native keratin have been classified as non-keratinolytic (21).

Keratinophilic fungi inhabit the soil, but have also been detected in the hair of mammals as well as bird feathers and nests (17, 18, 20). They include so-called geophilic dermatophytes as well as related species from the genus *Chrysosporium*. Three groups of species have been distinguished from the geophilic dermatophytes: a) non-pathogenic species represented primarily by *Trichophyton terrestre* Durie and Frey and *Trichophyton georgiae* Varsavsky and Ajello, b) frequently pathogenic species such as *Microsporum gypseum* (Bodin) Guiard and Grigoriakis and *Microsporum fulvum* Uriburu, and c) incidentally pathogenic species, including *Microsporum cookei* Ajello and *Trichophyton ajelloi* (Vanbreuseghem) Ajello, among others (32). Geophilic dermatophytes utilize the native keratin present in soil as a source of carbon, nitrogen, sulphur, and energy, and release large amounts of N-NH_4_ and S-SO_4_ as end-products (16). Therefore, they constitute an important group of decomposers of keratin residues in soil, which are a valuable nutritive substrate for these fungi and also permit their survival under conditions of nutrient competition with other saprotrophic microbial groups existing in the soil (9). The species *Trichophyton ajelloi* (formerly *Keratinomyces ajelloi*) was the first dermatophyte isolated from soil by hair-baiting, also referred to as the ToKoVa method (2, 31). In Europe, it is considered to be the dominant species that, as demonstrated by studies conducted in Germany and Poland, represents 60% of the total population of geophilic dermatophytes (19, 31). *T. ajelloi* strains are characterized by their strong keratinolytic capabilities. The extent of the utilization of native keratin (chicken feathers), as the sole source of C, N, and energy, by *T. ajelloi* in 21-day cultures varied from 50 to 76% depending on the particular strain (16).

Many molecular techniques have been applied for the identification and differentiation of different fungi species and strains (1, 8, 13, 14, 28, 29). In the present study, we used PCR-restriction fragment length polymorphism (PCR-RFLP) and microsatellite-primed PCR (MSP-PCR) to genetically characterize the *T. ajelloi* strains isolated in Poland from various soil samples. We used ITS1 and ITS4 universal primers for PCR amplification of the 5.8S rRNA gene and the neighboring two internal transcribed spacer regions (ITS1 and ITS2) (12), followed by *Hin*fI restriction enzyme digestion for molecular identification of the analyzed *T. ajelloi* collection. The PCR-RFLP method only produced species-specific fragments for the *T. ajelloi* isolates analyzed. In the second step, we used a repetitive (GACA)_4_ primer to examine the genetic diversity of *T. ajelloi* isolates inhabiting different soils. To the best of our knowledge, this is the first time that these molecular analyses have been applied to *T. ajelloi* populations.

## Material and Methods

### Soils

Keratinophilic fungi were isolated from 3 soils that differed typologically: a podzol, cambisol, and chernozem. The particle size distribution, content of macroelements, and pH of the soils included in this study are presented in Tables 1 and 2. Samples of the soils were taken once from 3 arable fields in the Lublin Region (central-eastern Poland). Podzol and cambisol soils were situated adjacent to each other (Sobieszyn 74.5 km from Lublin, 22°10′E 51°36′N). The chernozem soil was situated at a distance of 155 km from the other two soils (Grabowiec 94.1 km from Lublin, 23°33′E 50°49′N). Hence, the climate conditions for the podzol and cambisol soils were identical, while those for the chernozem soil were slightly different due to the distance from the other soils.

Soil samples were taken in spring (May) from fields under cereals. Approximately 25 g of soil was collected from each field from a depth of 1–20 cm (Ap horizon) at 20 uniformly distributed points to give a total of approximately 5–6 kg of soil. The samples were placed in sterile polyethylene bags. In the laboratory, the soil was mixed and screened through a sieve with a 2-mm mesh. After this preparation and stabilizing the soil moisture level to 50% of the total field capacity, 10 g of the soil samples were placed in 9-cm Petri dishes to half of their height. Fifty plates were prepared for each of the soils.

### Keratin substrate

The keratin substrate used in this study was white chicken (broiler) feathers, which were obtained from the Zakłady Drobiarskie “Indykpol” poultry company (Lublin, Poland). The feathers were washed, carefully rinsed with distilled water, dried, and then fragmented by cutting by hand into fragments of ca. 0.5 cm and sterilized in an autoclave (121°C, 1 atm., 30 min)

### Isolation and identification of keratinophilic fungi based on morphological traits

The isolation of keratinophilic fungi was conducted using the method of keratin bait, with chicken feathers as the substrate (15). Plates with the soil material were sprinkled with sterile feathers and incubated in a humid chamber at 26°C. After 4–6 weeks, the mycelia that appeared were inoculated onto Sabouraud glucose agar with actidione and chloramphenicol (7). The pure cultures obtained were kept on slants with Sabouraud medium (without antibiotics) at 4°C. Genus and species identification of the strains was performed on the basis of macroscopic observations on plates and microscopic observations in micro-cultures (Olympus BX-41 research microscope with a CVIII4 digital camera integrated with a computer with Cell-A software for image analysis, recording, and archiving). The final species classification based on morphological criteria was made according to the systematic studies by Domsch *et al.* (6), Oorschot (30), and Currah (5). The share of the populations of *T. ajelloi* in the assemblage of keratinophilic fungi in the particular soils was calculated as proportions, assuming that the total number of strains of keratinophilic fungi colonizing the plates with the studied soil was 100% and the number of strains of *T. ajelloi* was x %.

### Fungal strains used for molecular analysis

Seventy-five strains of *T. ajelloi* isolated from different soils were used for molecular identification and differentiation in this study (Table 3). Among them, 22 were isolated from podzol, 38 were isolated from cambisol, and 15 were isolated from chernozem. *T. ajelloi* CBS 119779, *T. rubrum* CBS 120358, and *T. mentagrophytes* CBS 120357 reference strains used in this study originated from Centraalbureau voor Schimmelcultures (Holland).

### DNA extraction

Total cellular DNA was extracted from a small amount of mycelia cultured on Sabouraud agar slants by a rapid mini-preparation method (23). The mycelia were added to 700 μL of lysis buffer (400 mM Tris-HCl, 60 mM EDTA, 150 mM NaCl, 1% sodium dodecyl sulphate), and incubated at 60°C for 1 h. After the addition of 210 μL of 3 M sodium acetate, the homogenate was centrifuged at 12,000 rpm for 15 min. The supernatant was successively extracted with phenol-chloroform-isoamyl alcohol (25:24:1). DNA was treated with RNase at a final concentration of 50 μg mL^−1^ for 20 min at 57°C. The samples were then precipitated using 3 volumes of cold ethanol in the presence of 300 mM sodium acetate and DNA was centrifuged for 10 min. The pellet was washed with 70% ethanol and air dried. DNA was dissolved in 30 μL TE buffer, and 1 μL of the resulting solution was used as a template in subsequent PCR reactions.

### PCR-RFLP identification

The ITS1, 5.8S, and ITS2 regions were amplified using the conserved primers ITS1 (5′-TCCGTAGTGGAACCTGCGG-3′) and ITS4 (5′ TCCTCCGCTTATTGATATGC-3′) (12). Each PCR mixture (30 μL) contained 1 μL of genomic DNA, 1 μL of 50 pmol of each primer, 12 μL of distilled water, and 15 μL of Taq PCR Master Mix Kit (Qiagen). Reaction mixtures were preheated to 95°C for 15 min, and 30 PCR cycles were then performed under the following conditions: 95°C for 1 min; 56°C for 1 min; and 72°C for 1 min. The thermal cycles were finalized by polymerization at 72°C for 10 min (29). PCR products were detected by electrophoresis in 1% agarose gel stained with ethidium bromide and visualized by UV light.

Seventy-five PCR products of the *T. ajelloi* strains and *T. ajelloi* reference strain, which were obtained using the ITS1 and ITS4 set of primers, were digested with the *Hin*fI (Fermentas) restriction enzyme for 2 h at 37°C, according to the manufacturer’s instructions. Digested fragments were separated by electrophoresis in an 8% polyacrylamide gel stained with ethidium bromide and visualized by UV light. Restriction profile analyses of the examined species were also performed by computer software (Vector NTI) based on DNA sequence data available in GenBank (NCBI, National Centre for Biotechnology Information, http://www.ncbi.nlm.nih.gov/genbank). The expected size of the DNA fragments from the genomic sequences of *Trichophyton ajelloi* (AJ000628) after the *Hin*fI digestion were 353 bp, 296 bp, and 8 bp.

### Microsatellite-primed PCR (MSP-PCR) using (GACA)_4_

MSP-PCR amplification was performed in a total reaction volume of 20 μL, containing 1 μL of genomic DNA (20 ng), 0.25 μL of 100 μM primer (GACA)_4_, 1 μL DMSO, 12.5 μL 2×PCR

MasterMix StartWarm (A&A Biotechnology), and 5.25 μL of distilled water. PCR was performed using the Thermal Cycler C1000 (Bio-Rad) as follows: initial denaturation at 95°C for 5 min, 40 cycles at 93°C for 60 s, 50°C for 60 s, 72°C for 60 s, and the final cycle at 72°C for 6 min (34). The PCR products were visualized under UV light after being separated by electrophoresis in a 1% agarose gel and stained with ethidium bromide.

### Data analysis

Strains with identical sizes and numbers of well-defined bands in the gels were considered to be genetically indistinguishable and were assigned to the same type. Strains with banding patterns that differed by up to two bands were considered to be closely related and were described as subtypes. Strains with banding patterns that differed by three or more bands were considered to be different types. Cluster analysis was performed using Versa Doc Imaging System version 1000 (Bio-Rad) with Quantity One software ( version 4.4.0). DNA relatedness was calculated by the band-based Dice coefficient with a setting of 2% band tolerance using the unweighted pair group method with mathematical averaging (UPGMA) (Fig. 2b).

The discriminatory power (D) of the MSP-PCR method using the (GACA)_4_ primer, calculated for *T. ajelloi* was determined using Simpson’s index diversity (11):

$$D=1-\frac{1}{N(N-1)}\sum_{j=1}^{s}n_{j}(n_{j-1})$$

where:

N—total number of strains of the tested species
S—total number of types (j)
n_j_—number of strains within a specific type (j).

The (*D*) value range was from 0 to 1. Values close to 1 indicated the high discriminatory power of the analyzed method.

The correlation index (r) (Table 4) was calculated to determine the relationship between the frequency of species of keratinophilic fungi and soil properties and also between genetic variations in the *T. ajelloi* isolates from various soils and the properties of the soils. Arstat software was used for calculations.

## Results

### Frequency of occurrence of *T. ajelloi* in the soils examined

The number of plates (samples) colonized by keratinophilic fungi was 36 (podzol), 38 (cambisol), and 44 ( chernozem), which constituted 72%, 76% and 88%, respectively. In total, 191 isolates of keratinophilic fungi: 39 from podzol, 55 from cambisol, and 97 from chernozem, and 182 isolates of non-keratinophilic fungi (were identified from the 3 soils unpublished data). The adopted rule was that one plate (sample) could be colonized by only one strain from a given species. Among the isolated keratinophilic fungi, we identified 7 species of non-dermatophyte fungi, which represented the group *Chrysosporium* (3), and a single species of a geophilic dermatophyte represented by *T. ajelloi*. In total, we isolated 75 strains of *T. ajelloi* from the soil samples analyzed (150 plates): 22 from podzol, 38 from cambisol, and 15 from chernozem, which constituted 61%, 100%, and 34%, respectively, of all keratinophilic fungi isolated from each of the soils examined. On average, *T. ajelloi* was found to inhabit 51% of the soil samples studied. The population of this dermatophyte represented 15% (chernozem), 56% (podzol), and 69% (cambisol) respectively, of the keratinophilic fungi communities of these soils.

### Correlations between the frequency of *Trichophyton ajelloi* and some physicochemical properties of the soils

In order to establish the reason for the intra-species differentiation of genotypes of *T. ajelloi* in the soils examined, coefficients of correlation (r) were calculated between the prevalence of *T. ajelloi* and some edaphic factors (Table 4). A correlation (*) was observed between *T. ajelloi* and the content of humus, total N, CaCO_3_, and pH. A negative coefficient of correlation for these pairs of features indicated an inverse relationship between the frequency of the fungus occurrence and these properties of the soil. It is noteworthy that the coefficient of correlation (*) obtained for the content of P_2_O_5_ was positive (Table 4).

### Molecular identification of *T. ajelloi* isolates

**Fig. 1:**
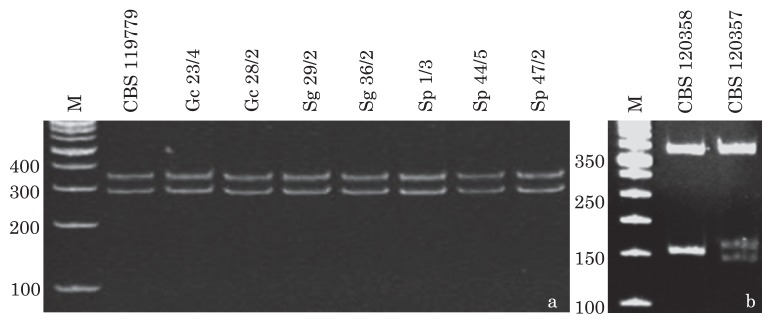
Exemplary polyacrylamide-gel electrophoresis of PCR products digested with the *Hin*fI restriction enzyme. The ITS1-ITS4 set of primers was used to amplify the ITS1-5.8SrDNA-ITS2 region. The profiles obtained for *Trichophyton ajelloi* (a), *Trichophyton rubrum* CBS 120358 (378, 154, 152, 8 bp), and *Trichophyton mentagrophytes* CBS 120357 (372, 158, 145, 8 bp) reference strains (b). Abbreviations above the lanes correspond to the species names assigned during traditional identification—Table 3.

The PCR products obtained during the amplification of DNAs from 75 *T. ajelloi* strains and the reference strain in the presence of the ITS1 and ITS4 (12) primers gave single bands of approximately 650 bp in each case. Comparative RFLP analysis using the *Hin*fI restriction enzyme (29) performed on all PCR products revealed only one pattern specific for *T. ajelloi* (Fig. 1a) and this could be distinguished from other species of *Trichophyton* (Fig. 1b).

### (GACA)_4_ typing

**Fig. 2:**
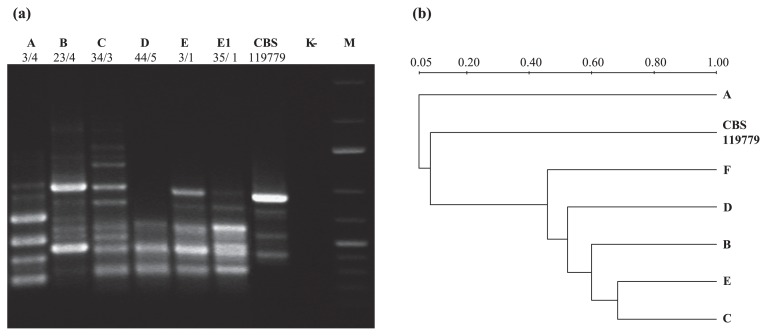
(GACA)_4_ genotyping of *T. ajelloi* strains (a) using the MSP-PCR method and unweighted pair group method with an arithmetic mean (UPGMA) dendrogram (b). Lane M, molecular size marker (2000, 1500, 1000, 700, 500, 400, 300, 200, 75 bp). Electrophoresis of the amplicons was carried out on a 1.2% agarose gel.

All of the examined *T. ajelloi* strains gave five characteristic band patterns (A–E) (Fig. 2a) after MSP-PCR amplification with the (GACA)_4_ primer. The obtained fingerprints yielded up to 12 bands, ranging from approximately 200 to 2,000 bp in length (Fig. 2a). Two patterns were distinguishable among the *T. ajelloi* strains isolated from podzol: A— which was characteristic for twelve isolates, and D—which was specific for ten isolates. In the thirty-eight strains of *T. ajelloi* isolated from cambisol, the most predominant genotype was C, represented by 22 strains, while genotype E (containing one subtype E1) was characteristic for 16 strains. All 15 isolates originating from chernozem were classified into one (GACA)_4_ genotype—B. The reference strain of *T. ajelloi* displayed a different genotype. None of the identified genotypes of *T. ajelloi* correlated with the soil properties being analyzed (content of humus, N total, CaCO_3_, fraction with ø <0.02 mm, and pH).

The discriminatory power of MSP-PCR using the (GACA)_4_ primer was very good, yielding Simpson’s index of diversity (*D*) at 0.823. All analyses were performed in duplicate, and PCRs were performed using two different thermal cyclers: Thermal Cycler C1000 (Bio-Rad) and Labcycler 48 Gradient (SensoQuest Biomedical Electronics). The duplicate MSP-PCR profiles showed an identical band pattern (data not shown) and the reproducibility was 100%.

## Discussion

Of the 150 soil samples (plates) examined, 79% were inhabited by keratinophilic fungi. In the soils included in this study, the group of geophilic dermatophytes was represented by just one species—*Trichophyton ajelloi*. In the ecological classification of keratinophilic fungi, *T. ajelloi* has been classified among the acidophilic fungi (10). This feature was also confirmed in the present study, in which the frequency of *T. ajelloi* was shown to increase with an increase in the acidity of the soil. The population of this geophilic dermatophyte was the largest in the acidic and strongly acidic (pH_KCl_ 4.4 and 3.4) cambisol and podzol, as evidenced by the presence of the fungus in 100% and 83% of the samples of these soils, respectively. Our earlier studies (19) also described the selection of populations of *T. ajelloi* in strongly acidic soils (pH_KCl_ 3.36; 4.06; 4.19; 4.29). In the present study, the preference of *T. ajelloi* for acidic and strongly acidic soils was also substantiated statistically. A preference for acidic soils on the part of *T. ajelloi* was also demonstrated by the negative coefficients of correlation between the frequency of that fungus and the content of CaCO_3_. Previous studies (4) did not isolate any keratinophilic fungi from the samples of strongly acidic soils (pH_KCl_ from 3.0 to 4.5). Marples (24) described *T. ajelloi* as an exceptionally acidophilic species that is only sporadically encountered in soils with pH>6.0. In the present study, a negative correlation was obtained between the frequency of occurrence of *T. ajelloi* and the content of humus and total nitrogen. This additionally demonstrated the stronger preference of this species for soils with lower levels of organic matter content. All *Trichophyton ajelloi* strains were analyzed using molecular methods, such as PCR-RFLP and MSP-PCR. The first method used in this study employed PCR to amplify the ITS region by using the ITS1 and ITS4 set of primers, followed by restriction analysis of the amplified products. Our results showed that digestion with the *Hin*fI restriction enzyme resulted in species-specific restriction profiles (Fig. 1). These results were consistent with the findings reported by Mochizuki *et al.* (28, 29), who reported that PCR-RFLP of the ITS region was a useful tool for the molecular identification of dermatophytes at the species level.

We used the MSP-PCR to determine whether it was possible to differentiate our isolates at the lower intra-species level. The (GACA)_4_ repetitive primer was chosen from the many primers described in the literature for the molecular typing of dermatophytes (13, 14). Faggi *et al.* (8) described the application of PCR for the molecular differentiation of clinical dermatophyte species and strains using the repetitive oligonucleotide (GACA)_4_ as a primer, which was previously used successfully by Meyer *et al.* (26, 27) to distinguish strains of *Cryptococcus neoformans* and *Candida* species. The findings obtained by Faggi *et al.* (8) showed that there was no intra-species variability in the case of the analyzed *M. canis* strains isolated from humans and animals (cats and dogs) in Spain. The same findings were obtained by Shehata *et al.* (34) who analyzed *M. canis* strains isolated from Egyptian patients and also identified only one genotype among them. These findings suggest that the (GACA)_4_ primer may have a low differentiation power for clinical strains of dermatophytes. In the present study, we analyzed *T. ajelloi* strains, a geophilic dermatophyte with a worldwide distribution that can occur as a saprophytic contaminant on humans and animals (32). In the case of this group of dermatophytes, we noted that the discriminatory power of the (GACA)_4_ primer was high (Simpson’s index of diversity D = 0.823). Our (GACA)_4_ typing results showed five genotypes (A–E) among the 75 analyzed strains of *T. ajelloi* isolated from podzol—genotypes A and D, cambisol—genotypes C and E (containing one subtype E1), and chernozem—genotype B (Fig. 2a). The *T. ajelloi* strains originating from cambisol and podzol, strongly acidic and acidic soils, gave two patterns (Fig. 2a) (Table 3), which suggests that the abundance and diversity of *T. ajelloi* may be higher than those in chernozem— a soil with a neutral reaction. The results of our study revealed that the species analyzed, apart from a very high (100%) or high frequency (61%) of occurrence in strongly acidic soils, also appeared in chernozem (in 34% of the soil samples), *i.e.* a soil with a neutral reaction (pH_KCl_ 7.15). This may suggest selection of *T. ajelloi* strains which are more tolerant to changes in pH, and hence their genotype is different from those which are represented in populations of *T. ajelloi* isolated from acidic soils (cambisol, podzol). The genetic homogeneity of the strains of *T. ajelloi* colonizing chernozem may be related to the original occurrence in these soils (steppe chernozems) of *Micromammalia*, which is a source of a specific type of keratin (hair keratin) (18). However, the selection of a greater number of genotypes within the *T. ajelloi* populations in cambisol and podzol may be attributed to the generally unfavourable conditions for keratinophilic fungi prevailing in those soils. Most keratinophilic fungi have a preference for soils characterized by a notable content of organic matter, especially of animal origin, a close to neutral reaction, and a favorable air-water relationship. Such soils include chernozems (17, 18). The soils used were characterized by high acidification and excessive (podzol) or too low (cambisol) permeability, and, as a consequence, by a negative water balance or insufficient amount of air as well as a low or very low (podzol) content of organic matter. In this context, the greater diversity of genotypes within the *T. ajelloi* populations in both these soils may be due the adaptation effects to the unfavourable conditions. On the other hand, the lack of correlations between genetic variations in the strains of *T. ajelloi* and the physicochemical parameters of the soils examined may indicate that the genetic diversity of the species is determined by the entirety of features composing a specific type of soil and not by the individual properties of the soils. Therefore, the results of our study permit the conclusion that various types of soil are colonized by genotype-diverse populations of *T. ajelloi*.

The MSP-PCR method of DNA fingerprinting is now commonly used for dermatophytes isolated from humans and animals (1, 13, 14, 28). Our preliminary results indicate that the (GACA)_4_ repetitive primer, which was employed in geophilic dermatophyte typing for the first time in the present study, could be used to subtype *T. ajelloi*. However, these results should be confirmed after checking other primers and methods of differentiation. In future studies, we intend to apply an alternative method, such as a PCR melting profiles (PCR-MP) technique based on the method of ligation-mediated PCR (25), to the intra-species differentiation of *T. ajelloi* and other geophilic dermatophytes. This method has been successfully implemented by our group for clinical strains of *T. rubrum*, *T. interdigitale*, and *M. canis* isolated from patients in Poland (22).

## Figures and Tables

**Table 1:** Particle size distribution of the soils included in this study

Soil type-locality	Mechanical formation	Percentage of fraction of ø in mm								

		>1.0	1.0–0.5	0.5–0.25	0.25–0.1	0.1–0.05	0.05–0.02	0.02–0.006	0.006–0.002	<0.002
Chernozem-Grabowiec^a^	Loess	n.p.	—	—	—	11.0	47.0	23.0	10.0	7.0
Podzol-Sobieszyn^b^	Loamy sand	n.p.	6.0	31.0	41.0	7.0	8.5	1.5	3.0	2.0
Cambisol-Sobieszyn^c^	Heavy clay	n.p.	8.0	13.5	23.5	10.5	12.5	8.5	7.0	16.5

Fraction of ø<0.02 in mm ^a^ 40.0; ^b^ 6.5; ^c^ 32.0.

Explanations: n.p., not present; —, not examined.

**Table 2:** Selected chemical properties of the soils included in this study

Soil type-locality	humus	N tot.	CaCO_3_	P_2_O_5_ mg in 100 g of soil	pH w KCl

	%				
Chernozem-Grabowiec	3.93	0.270	0.23	13.3	7.15
Podzol-Sobieszyn	0.35	0.042	0.00	12.4	3.4
Cambisol-Sobieszyn	1.26	0.098	0.00	16.6	4.4

**Table 3:** (GACA)_4_ types of *T. ajelloi* strains isolated from soils in Poland

Source of strains (soil type)	Total number of isolates	(GACA)_4_ type					

		A	B	C	D	E	E1
Podzol	22	12	0	0	10	0	0
Chernozem	15	0	15	0	0	0	0
Cambisol	38	0	0	22	0	11	5

**Table 4:** Correlation coefficients (r) between the frequency of *T. ajelloi* and certain properties of the soils examined (at a significance level of α=0.05)

humus	N-total	CaCO_3_	P_2_O_5_	pH	fraction (Ø<0.02 mm)
−0.5463	−0.5403	−0.7346	0.8745	−0.5351	−0.085
